# Compatible topologies and parameters for NMR structure determination of carbohydrates by simulated annealing

**DOI:** 10.1371/journal.pone.0189700

**Published:** 2017-12-12

**Authors:** Yingang Feng

**Affiliations:** 1 Shandong Provincial Key Laboratory of Synthetic Biology, Qingdao Institute of Bioenergy and Bioprocess Technology, Chinese Academy of Sciences, Qingdao, China; 2 CAS Key Laboratory of Biofuels, Qingdao Institute of Bioenergy and Bioprocess Technology, Chinese Academy of Sciences, Qingdao, China; 3 Qingdao Engineering Laboratory of Single Cell Oil, Qingdao Institute of Bioenergy and Bioprocess Technology, Chinese Academy of Sciences, Qingdao, Shandong, China; National Cancer Institute at Frederick, UNITED STATES

## Abstract

The use of NMR methods to determine the three-dimensional structures of carbohydrates and glycoproteins is still challenging, in part because of the lack of standard protocols. In order to increase the convenience of structure determination, the topology and parameter files for carbohydrates in the program Crystallography & NMR System (CNS) were investigated and new files were developed to be compatible with the standard simulated annealing protocols for proteins and nucleic acids. Recalculating the published structures of protein-carbohydrate complexes and glycosylated proteins demonstrates that the results are comparable to the published structures which employed more complex procedures for structure calculation. Integrating the new carbohydrate parameters into the standard structure calculation protocol will facilitate three-dimensional structural study of carbohydrates and glycosylated proteins by NMR spectroscopy.

## Introduction

Carbohydrates are important molecules for energy storage and metabolism in all organisms. Carbohydrate binding proteins play roles in carbohydrate biosynthesis, transport, metabolism, and degradation. Further, glycosylation is an important protein post-translational modification that affects protein stability, conformation and signaling function. NMR structures of proteins and nucleic acids account for about 10% of the total number of structures in the Protein Data Bank (PDB) [1], but NMR structures of carbohydrate-protein complexes, glycosylated proteins, and solo carbohydrate molecules represent only a small fraction of these. As of June 2017, there were more than 11,000 solution NMR structures in the PDB, but of these only about 50 structures contain saccharides. The large molecular weight and poor dispersion of sugar NMR signals can cause difficulties in NMR structure determination. However, a key factor contributing to the low number of carbohydrate-containing structures is likely to be the lack of a simple and convenient protocol including topologies and parameters to determine the NMR structure of carbohydrate molecules.

NMR methods have been employed to investigate saccharides, providing rich structural information, such as anomeric configuration, molecular weight, conformation and dynamics. Due to the flexible nature of carbohydrates and their poor chemical shift dispersion, NMR structure determination is often carried out in conjunction with molecular modelling [2]. Researchers have made great efforts to develop force fields for carbohydrates, and the GLYCAM force field is the most widely used [3]. In addition, most of the popular molecular dynamics programs, such as Amber, GROMACS and CHARMM, have their own carbohydrate force fields [4–6]. However, for NMR structure determination of biomacromolecules, specialized programs are often used to combine the experimental restraints with the chemical structure constraints (i.e. structural parameters). The chemical structure constraints are defined in topology and parameter files. The topology file contains the definition of residues (units of biopolymers) and patches which make specific chemical modifications to residues (such as disulfide bonds). The topology definition includes the terms BOND (the chemical bond length between two atoms), ANGLE (the bond angle of two bonds connected to one atom), DIHEDRAL (the dihedral angle formed by four atoms, which is generally rotatable between conformations), and IMPROPER (the dihedral angle formed by four atoms which need not correspond to a rotatable bond, used in NMR structure calculations to maintain proper chirality of a tetrahedral center or to maintain planarity in a conjugated region, and thus is generally held rigid). The parameter file contains the values and energies of each type of topology term in the topology file. The NMR programs sacrifice part of the accuracy of the force field to obtain the compact three-dimensional structure from a linear extended structure within a reasonable calculation time frame. The calculation protocols in these programs are generally convenient and are widely used by researchers, providing reliable and comparable structures.

The most popular and widely used programs for NMR structure calculations include Crystallography & NMR system (CNS) [7], XPLOR/Xplor-NIH [8], and DYANA/CYANA [9, 10]. CNS and XPLOR/Xplor-NIH have almost identical file formats for topology and parameters, and both contain carbohydrate files designed for X-ray structure determination which are not well validated for NMR structure calculation. DYANA/CYANA does not contain topology and parameter files for carbohydrates. The literature describing NMR structures of carbohydrates provides several approaches to overcome the lack of topology and parameter files. One way is to use other generic molecular dynamics software at the refinement stage that can handle carbohydrates [11, 12], such as Amber [4]. Another way is to use parameters generated by other software or web-servers that are designed for the generation of parameters for any type of ligand, such as XPLO2D [13] and the PRODRG server [14]. There are also other approaches, such as modification/extension of original topology/parameter files for CYANA, CNS or XPLOR/XPLOR-NIH by combining with other programs [15–17]. However, the reported procedures often lack sufficient detail to be readily reproducible by other researchers. In addition, these combined methods complicate the structure determination process, and sometimes require specialist protocols that are different from the well-established protocols for proteins and nucleic acids [18]. Therefore, there is a need to construct convenient and compatible carbohydrate parameters that can be used with the well-established protocols for protein and nucleic acid structure calculation.

In this study, the carbohydrate topology and parameter files for CNS were checked and modified. The modifications were kept to a minimum and were verified to be compatible with the simulated annealing protocols for proteins and nucleic acids. The modifications include: the energies of BOND, ANGLE, DIHEDRAL, and IMPROPER terms were set to the corresponding values for protein/nucleic acid parameters; IMPROPER term definitions in the topology file were revised for sugar ring chiral centers; patches for O- and S-glycosylation were added; and some of the missing patches for L-saccharides as well as other missing parameters were also added. The new topology and parameter files were validated by recalculation of previously published structures including protein-carbohydrate complexes and glycosylated proteins.

## Materials and methods

### Topology and parameter files

The original topology and parameter files carbohydrate.top and carbohydrate.param in CNS version 1.3 were modified. First, three parameters for the IMPROPER term of the NAG N2-C7 peptide bond were added to prevent the CNS program from terminating during the structure calculation process due to errors. Second, the energies for the BOND, ANGLE, IMPROPER, and DIHEDRAL terms were set to 1000.0, 500.0, 500, and 2.0, respectively, which are the same as the corresponding values for protein/nucleic acid parameters (protein-allhdg5-4.top and protein-allhdg5-4.param). Third, the IMPROPER terms of all sugar chiral carbons were redefined using four tetrahedral vertex atoms. Fourth, a number of patches were constructed for O- and S-glycosylation with α- or β-linkages at C1 for both L- and D-saccharides. The new topology and parameter files (carbohydrate-nmr.top and carbohydrate-nmr.param) are provided in the S1 File of this paper.

### Structure calculation

The experimental restraints of the structures containing saccharides were downloaded from BioMagResBank (http://www.bmrb.wisc.edu) [19] or the Protein Data Bank [1]. The standard protocols in the files generate_seq.inp and generate_extended.inp for CNS were used to generate a linear extended structure as the initial structure for the simulated annealing calculations. The structures were recalculated using the default simulated annealing protocol (in the file anneal.inp) in CNS version 1.3. For each protein, a total of 100 structures were calculated and the 20 lowest energy structures were selected for analysis. The structures were visualized and analyzed using the program PyMol (http://www.pymol.org) (Schrödinger, LLC). Carbohydrate Ramachandran plots were generated using the CARP server (http://www.glycosciences.de/tools/carp/) [20].

## Results and discussion

**Fig 1:**
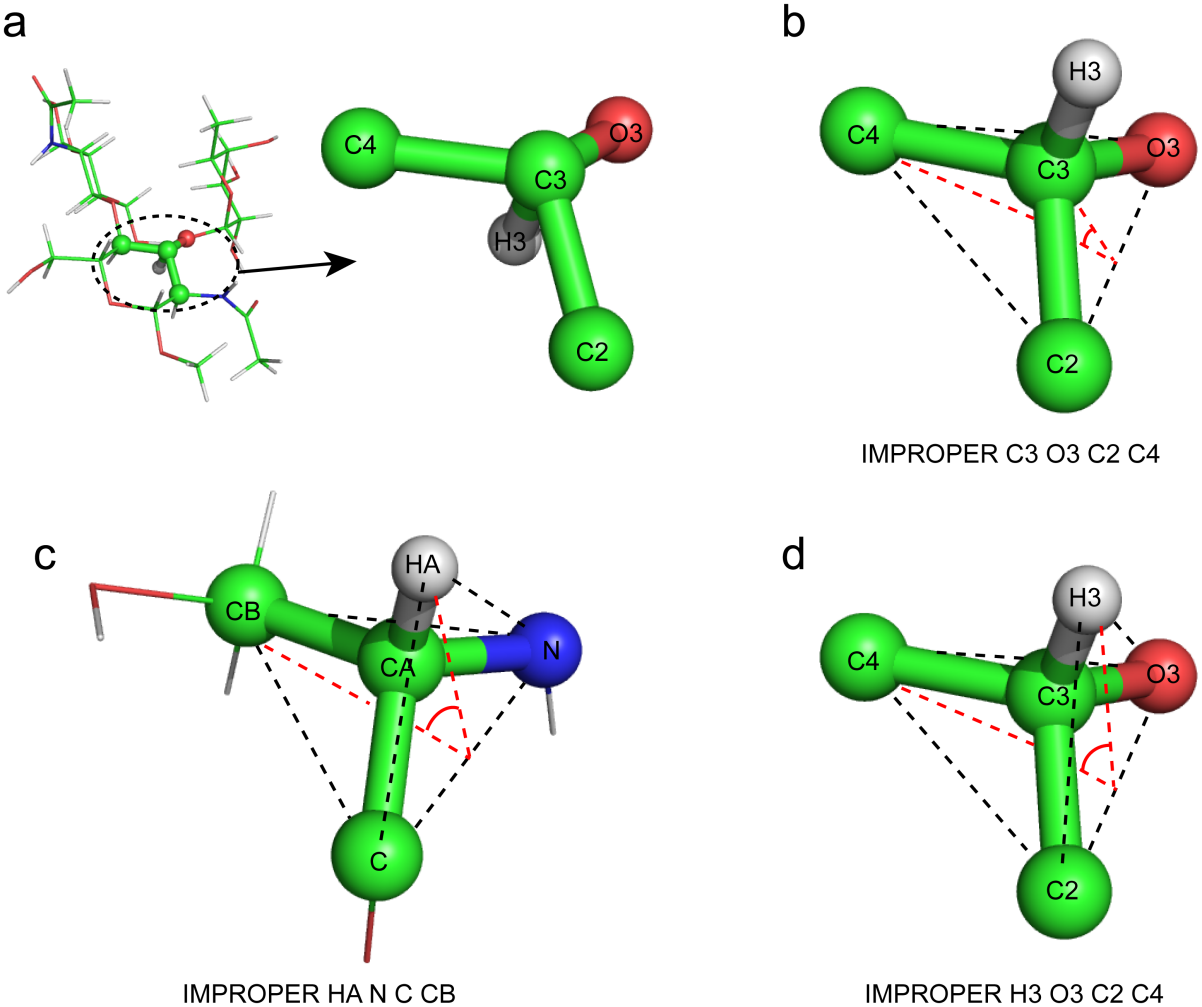
The problem of IMPROPER term definition for the chiral carbon atom. **a**, The problematic structure of the sugar ring calculated from the original carbohydrate topology file. **b**, The definition of the IMPROPER term of the sugar ring chiral carbon atom C3 in the original carbohydrate topology file. **c**, The definition of the IMPROPER term of serine CA atom in the protein topology file. **d**, The new definition of the IMPROPER term of the sugar chiral carbon atom C3.

To check the original carbohydrate topology and parameter files in CNS, I recalculated a previously published structure of CCL2 in complex with a glycan (PDB 2LIQ) [11]. The published structure was originally calculated using CYANA and was refined in Amber with NOE-derived distance restraints, hydrogen bond distance restraints, and backbone dihedral angle restraints. In my calculation, the same restraints were used in the default simulated annealing protocol of CNS. I found that the CNS program terminated due to errors because the original files carbohydrate.top and carbohydrate.param lacked three IMPROPER term parameters for the NAG N2-C7 peptide bond. After adding the missing parameters, I also found that the energies for the BOND, ANGLE, IMPROPER, and DIHEDRAL terms in the carbohydrate parameter file were significantly different from those in the protein/nucleic acid parameter files. Some of the ANGLE term energies in the carbohydrate parameter file were smaller than those for experimental restraints in the default simulated annealing protocol of CNS, which will result in large angle deviations from the ideal geometry in the calculation. Therefore, I recalculated the structures after setting the energies of BOND, ANGLE, IMPROPER, and DIHEDRAL terms in the carbohydrate parameter file to the same values as those in the protein/nucleic acid parameter files. However, when I inspected the conformation of the carbohydrate, some chiral carbon atoms in the sugar ring have problematic bonding linkages (Fig 1a). Each of these carbon atoms has one bonded hydrogen atom whose position is incorrect because of flipping along the line of the C-H bond and the carbon center. Since the carbon chirality in the original topology file carbohydrate.top is defined by the IMPROPER term of the central carbon and the surrounding heavy atoms (i.e. non-hydrogen atoms) (Fig 1b), there is no energy for the IMPROPER term applied to the hydrogen atom. The position of the hydrogen atom was therefore restrained only by BOND and ANGLE parameters, and if the overall energy was minimized, this should be sufficient to drive the hydrogen atom to the correct position. However, without an energy value for the IMPROPER term applied to the hydrogen atom, the only energy change among all energy terms is the BOND energy, which is defined by the non-directional distance between two atoms when the hydrogen atom moves along the line connecting the central atom and the ideal tetrahedral vertex during the simulated annealing. This local energy minimum will result in trapping of the molecule in the flipped position, which caused the problem observed in the simulated annealing of the carbohydrate. In crystallography, this problem will not arise, because hydrogen atoms are not added at low resolution, and at high resolution, the electron density will provide additional constraints to avoid the flipping.

**Fig 2:**
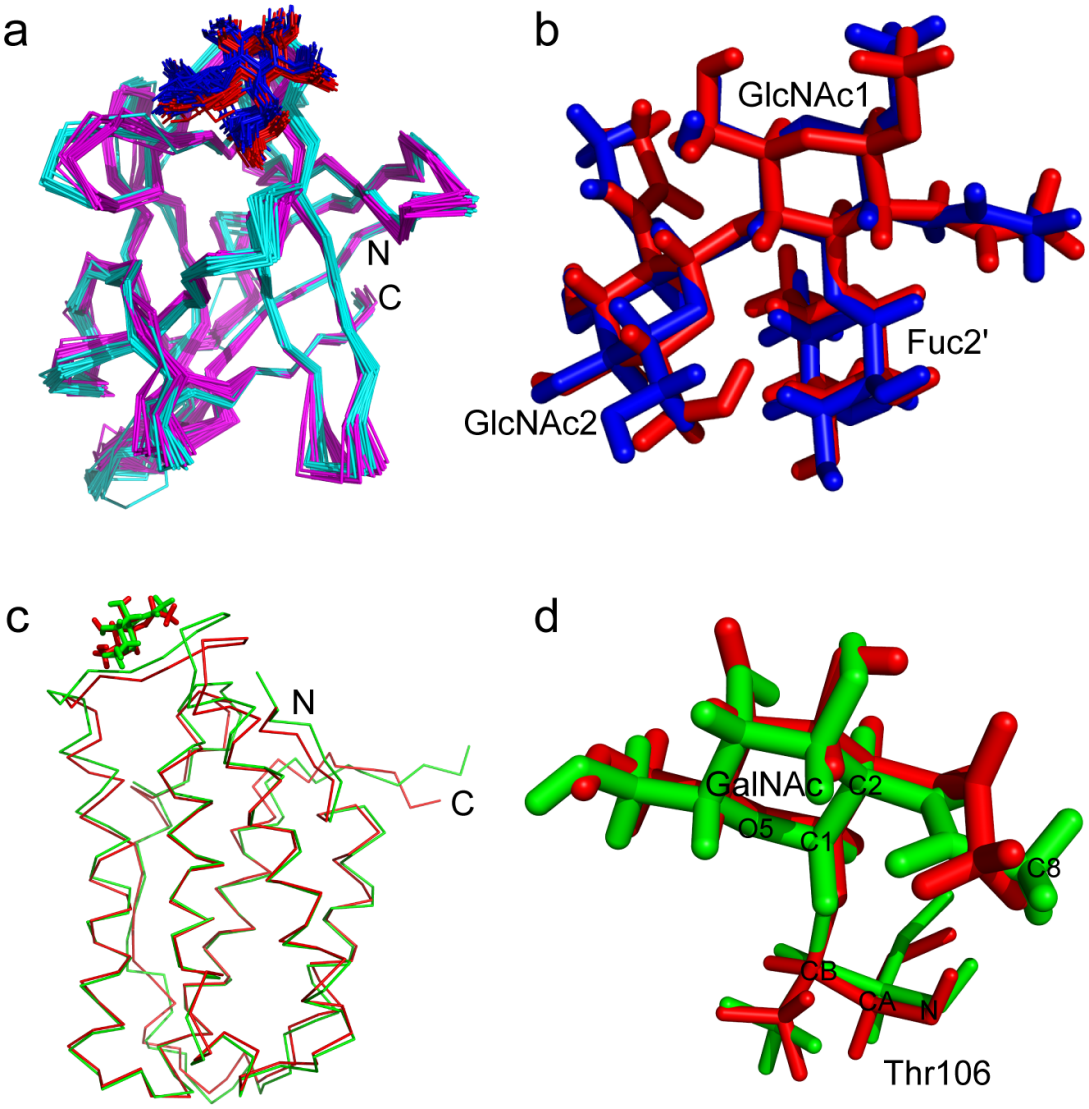
Comparison of the original structures and the recalculated structures using the new topology and parameter files. **a**, The structures of the CCL2-glycan complex. Magenta and cyan, the backbone ribbon representation of the protein part in the original (PDB 2LIQ) and recalculated structures, respectively; red and blue, the stick representation of the glycan part in the original and recalculated structures, respectively. **b**, The close-up view of the glycan structures. The colors are the same as in **a**. **c**, The structures of the O-linked glycoprotein GalNAcα-IFNα2a. Since the glycosylation site is located in a partially flexible loop, only one structure is shown for clarity. Red, original structure; green, recalculated structure. The proteins are shown as backbone ribbons, while the sugar residues are shown as sticks. **d**, The close-up view of the glycosylated threonine structures. The colors are same as in **c**. For clarity, some atoms are labeled.

**Fig 3:**
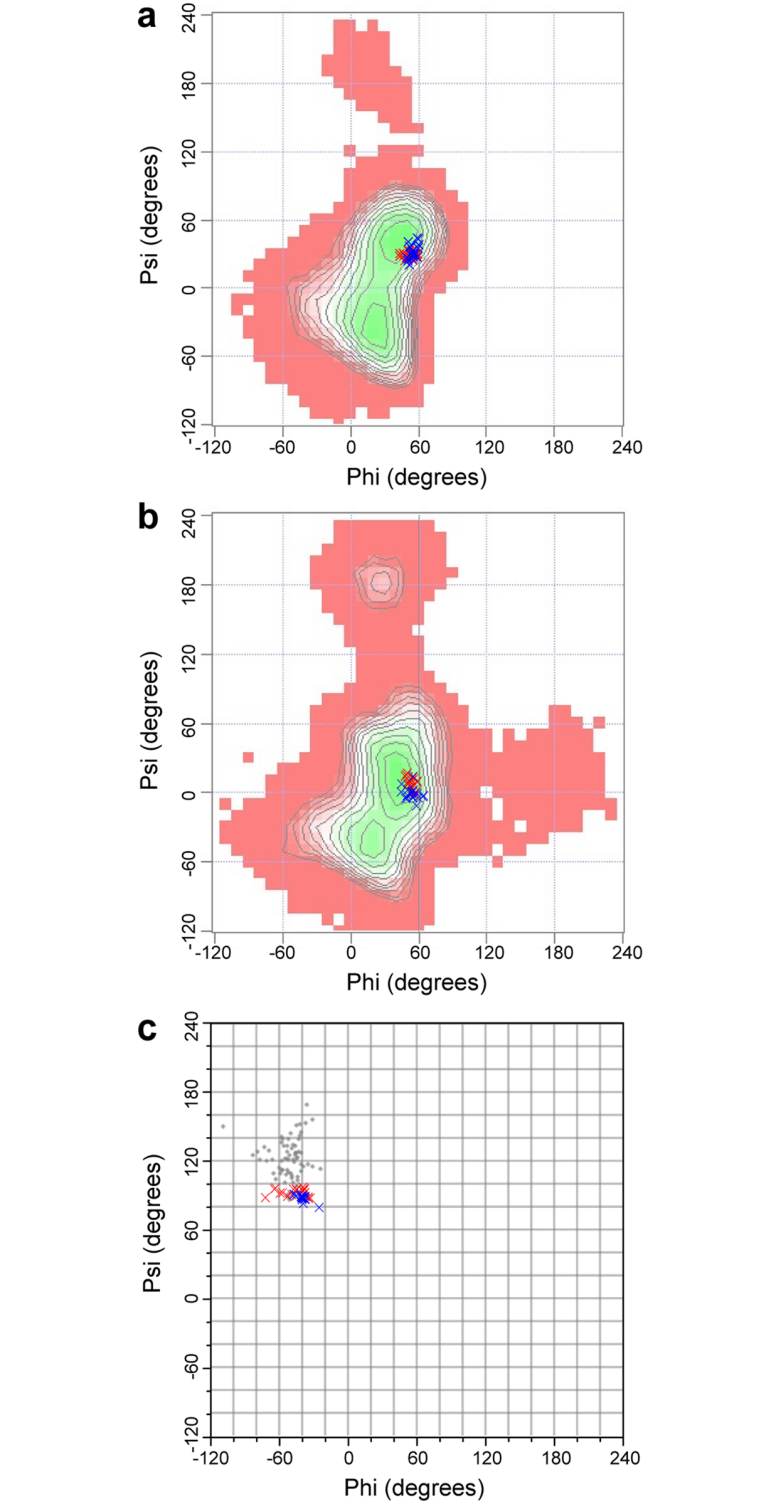
The carbohydrate Ramachandran plots of original and recalculated structures. Red crosses, original structures; blue crosses, recalculated structures. **a**, The plot of α-Fuc-(1,3)-β-GlcNAc torsion angles in the structures of the CCL2-glycan complex. **b**, The plot of β-GlcNAc-(1,4)-β-GlcNAc torsion angles in the structures of the CCL2-glycan complex. **c**, The plot of α-GalNAc-Thr torsion angles in the structures of the O-linked glycoprotein GalNAcα-IFNα2a. The background plots in **a** and **b** are the maps from GlycoMapsDB (http://www.glycosciences.de/modeling/glycomapsdb/), while the background plot in **c** is the torsion angles from PDB (grey dots) because of no α-GalNAc-Thr map in GlycoMapsDB.

The protein part of the structure has no hydrogen flipping problem of chiral carbon atoms in the simulated annealing calculation, and the chirality of all the chiral carbons in the protein is defined by the IMPROPER term energies of the four tetrahedral vertex atoms surrounding the central carbon atom in the protein topology file (Fig 1c). This definition provides the same energy for the four atoms, so there is no local energy minimum in the flipped position. Therefore, I modified the carbohydrate topology and parameter files, and replaced the IMPROPER term definition of the sugar ring chiral carbon atoms using the four tetrahedral vertex atoms including hydrogen atoms (Fig 1d). After recalculation using the newly defined topology and parameter files, the structures of the CCL2-glycan complex showed the correct chiral carbon bonding, and the structures are very similar to the published structures (Fig 2a and 2b). The carbohydrate Ramachandran plots of original and recalculated structures show a similar phi-psi torsion angle distribution in the favored region (Fig 3a and 3b; Table 1). This indicates that the new topology and parameter files are compatible with the standard simulation annealing protocol in the NMR structure calculation, and the results are comparable with other molecular dynamics software such as Amber. It should be noted that the energy of the DIHEDRAL term is much smaller than the other three term energies in both the original and the new modified parameter files, because dihedral angles between conformations are variable over a wide range, which is different from the BOND, ANGLE, and IMPROPER terms. Therefore, the qualitative uniform small DIHEDRAL energy is used only to simplify the parameters, and is not critical for the structure calculation.

**Table 1:** The torsion angles of carbohydrates in the original and recalculated structures.

	Phi (original)	Phi (recalculated)	Psi (original)	Psi (recalculated)
**α-Fuc-(1,3)-β-GlcNAc of PDB 2LIQ**	53.4 ± 4.7	55.9 ± 3.5	29.3 ± 2.5	32.0 ± 6.7
**β-GlcNAc-(1,4)-β-GlcNAc of PDB 2LIQ**	53.1 ± 3.1	54.0 ± 5.3	8.35 ± 5.1	-1.0 ± 5.2
**α-GalNAc-Thr of PDB 2LMS**	-46.5 ± 11.4	-39.7 ± 4.5	92.7 ± 3.2	87.9 ± 2.6

The original carbohydrate topology and parameter files have no patch for O-glycosylation and S-glycosylation, although O-glycosylation is a frequent post translational modification in proteins. Some patches for the linkages of L-saccharides are also absent. In my newly developed topology file, I construct the patches A1S and A1T for α-linkage from the carbohydrate C1 to Ser OG and Thr OG1, B1S and B1T for β-linkage from the carbohydrate C1 to Ser OG and Thr OG1, A1C and B1C for α- and β-linkage from the carbohydrate C1 to Cys SG, B13 and B16 for β(1,3)- and β(1,6)-linkages, and B13L, B16L, A1SL, B1SL, A1TL, B1TL, A1CL and B1CL for the linkages of L-saccharides. The new patches for O-glycosylation were tested by recalculating the structure of the glycoprotein GalNAcα-IFNα2a (PDB 2LMS) which contains an O-glycosylated threonine [16]. In the GalNAcα-IFNα2a structure, the carbohydrate is more flexible than most parts of the protein. There are only 17 restraints between the carbohydrate A2G and the protein, and they are all on the A2G H1 and H8 atoms. In the recalculated structures, all carbohydrate carbons have the correct bonding and chirality. The sugar rings of about half of the structures present a chair conformation, while others present boat or envelope conformations. In the published structures, all the sugars are in the chair conformation; however, in the structure calculation there is insufficient conformational restraint (NOE or dihedral) applied to achieve the chair conformation. Nevertheless, the chair conformation is the most stable conformation of pyranose in general, so the sugar is generally assumed to be in the chair conformation in the absence of experimental evidence to suggest otherwise [18, 21]. NMR analysis of J-couplings can also be used to experimentally determine the conformation of the sugar ring [2]. In either case, additional dihedral restraints on the sugar ring can be introduced in the structure calculation. I performed a new calculation of PDB 2LMS with two additional dihedral restraints to the sugar ring, resulting in structures which are very similar to the published ones (Fig 2c and 2d). The carbohydrate Ramachandran plots of the original and recalculated structures also show similar phi-psi torsion angle distributions (Fig 3c and Table 1). This demonstrates that the topology and parameters presented here can be used to determine the structures of glycosylated proteins with the standard simulated annealing protocol in CNS. These topology and parameter files were also used in the recent structure determination of several O-glycosylated carbohydrate binding modules, providing structural insight into the stabilizing effect of O-glycosylation [22].

The topology and parameter files were further tested by recalculating most of the protein-carbohydrate complex and glycosylated proteins available in the PDB. These structures include four protein-carbohydrate complexes, seventeen glycosylated proteins/peptides, and one carbohydrate (Table 2). These calculations demonstrate that the new topology and parameter files for carbohydrates work well with the standard CNS simulated annealing protocol for different kinds of protein and carbohydrate molecules. It should be noted that, similar to PDB 2LMS, the sugar rings in the calculated structure ensemble may present different conformations if the carbohydrate is flexible or lacks sufficient distance restraints, while in some previous studies, additional dihedral angle restraints were introduced to maintain the sugar ring in the theoretical lowest energy conformation or the conformation observed in crystal structures [21, 23, 24].

**Table 2:** Tested structures in this study: The RMSD of sugar heavy atoms are compared.

	PDB numbers	RMSD-O ^a^ (Å)	RMSD-R ^b^ (Å)	RMSD O-R ^c^ (Å)
**Protein-carbohydrate complex**	1ACZ	6.86	5.54	6.88
	2JXA ^d^	0.78	1.49	1.63
	2LIQ	0.46	0.94	0.80
	2KR2	0.10	2.06	2.43
**Glycosylated protein**	1AH1	3.49	2.10	3.95
	1FF7	0.001	0.004	0.059
	1KYJ	0.70	1.16	2.06
	2JXA ^d^	0.78	1.49	1.63
	2LHW	1.37	2.67	2.24
	2LHX	0.74	1.24	1.79
	2LHY	1.06	1.75	2.08
	2LHZ	0.49	0.91	1.58
	2LI0	0.13	0.43	0.40
	2LI1	0.12	0.40	0.41
	2LI2	0.11	0.44	0.47
	2LMS	0.60	0.47	0.98
	2MIJ	0.001	0.47	0.47
	2MK7	0.79	1.58	1.46
	2RQZ	0.41	0.63	0.52
	2RR2	0.0024	0.0025	0.013
	4B1Q	1.23	0.51	1.11
**Carbohydrate**	2MK1	0.06	0.36	0.62

^a^ The RMSD of sugar heavy atoms in the original structures.

^b^ The RMSD of sugar heavy atoms in the recalculated structures.

^c^ The RMSD of sugar heavy atoms between the original and recalculated structures.

^d^ The PDB 2JXA structure is both glycosylated and contains a bound carbohydrate.

For most of the tested structures, it should be noted that the RMSDs in the recalculated structures are larger than those in the original structures. The larger RMSDs suggest that the recalculated structures are more “flexible” than the original, which may be caused by differences in the force field, calculation protocol, and/or by the lack of a refinement stage in the recalculation. However, some extremely large differences indicate that some published structures were over-refined by employing an MD program/force field. For example, in the structure of 2KR2, the sugar (maltose) has a very small RMSD (0.10 Å) after refinement using the COSMOS field [15], but the sugar binds to a partially flexible loop region with an RMSD of ~2.5 Å. There are some NOE restraints between the sugar and protein, but no intramolecular NOE restraint for the sugar was used. Therefore, the extremely low RMSD of the sugar arises from the refinement, which resulted in a single conformation in the published structures. In my recalculated structures, the RMSD becomes 2.06 Å, which is more reasonable for a sugar that binds to loops with ~2.5 Å RMSD.

In summary, I have presented a suite of CNS topology and parameter files for carbohydrates. These files were demonstrated to be compatible with the standard simulated annealing calculation protocols for proteins and nucleic acids. Because CNS and XPLOR/Xplor-NIH share similar topology/parameter file format and the file modification procedures are simple, these files could be easily integrated into future upgrades of these programs. Integrating the carbohydrate topology and parameters into the standard structure calculation protocol will facilitate the three-dimensional structural study of carbohydrates and glycosylated proteins by NMR spectroscopy.

## Supporting information

S1 File Revised carbohydrate topology and parameter files. (ZIP)

## References

[1] Berman HM, Westbrook J, Feng Z, Gilliland G, Bhat TN, Weissig H, et al The Protein Data Bank. Nucleic Acids Res. 2000;28(1):235–242. doi: 10.1093/nar/28.1.235 10592235 10.1093/nar/28.1.235 PMC102472

[2] Diaz D, Canales-Mayordomo A, Canada FJ, Jimenez-Barbero J. Solution conformation of carbohydrates: a view by using NMR assisted by modeling. Methods Mol Biol. 2015;1273:261–287. doi: 10.1007/978-1-4939-2343-4_19 25753717 10.1007/978-1-4939-2343-4_19

[3] Kirschner KN, Yongye AB, Tschampel SM, Gonzalez-Outeirino J, Daniels CR, Foley BL, et al GLYCAM06: A generalizable biomolecular force field. Carbohydrates. J Comput Chem. 2008;29(4):622–655. doi: 10.1002/jcc.20820 17849372 10.1002/jcc.20820 PMC4423547

[4] Case DA, Cheatham TE, Darden T, Gohlke H, Luo R, Merz KM, et al The Amber biomolecular simulation programs. J Comput Chem. 2005;26(16):1668–1688. doi: 10.1002/jcc.20290 16200636 10.1002/jcc.20290 PMC1989667

[5] Van der Spoel D, Lindahl E, Hess B, Groenhof G, Mark AE, Berendsen HJC. GROMACS: Fast, flexible, and free. J Comput Chem. 2005;26(16):1701–1718. doi: 10.1002/jcc.20291 16211538 10.1002/jcc.20291

[6] Guvench O, Mallajosyula SS, Raman EP, Hatcher E, Vanommeslaeghe K, Foster TJ, et al CHARMM additive all-atom force field for carbohydrate derivatives and its utility in polysaccharide and carbohydrate-protein modeling. J Chem Theory Comput. 2011;7(10):3162–3180. doi: 10.1021/ct200328p 22125473 10.1021/ct200328p PMC3224046

[7] Brunger AT, Adams PD, Clore GM, DeLano WL, Gros P, Grosse-Kunstleve RW, et al Crystallography & NMR system: A new software suite for macromolecular structure determination. Acta Crystallogr Sect D Biol Crystallogr. 1998;54:905–921. doi: 10.1107/S0907444998003254 9757107 10.1107/s0907444998003254

[8] Schwieters CD, Kuszewski JJ, Tjandra N, Clore GM. The Xplor-NIH NMR molecular structure determination package. J Magn Reson. 2003;160(1):65–73. doi: 10.1016/S1090-7807(02)00014-9 12565051 10.1016/s1090-7807(02)00014-9

[9] Guntert P, Mumenthaler C, Wuthrich K. Torsion angle dynamics for NMR structure calculation with the new program DYANA. J Mol Biol. 1997;273(1):283–298. doi: 10.1006/jmbi.1997.1284 9367762 10.1006/jmbi.1997.1284

[10] Guntert P, Buchner L. Combined automated NOE assignment and structure calculation with CYANA. J Biomol NMR. 2015;62(4):453–471. doi: 10.1007/s10858-015-9924-9 25801209 10.1007/s10858-015-9924-9

[11] Schubert M, Bleuler-Martinez S, Butschi A, Walti MA, Egloff P, Stutz K, et al Plasticity of the beta-trefoil protein fold in the recognition and control of invertebrate predators and parasites by a fungal defence system. PLoS Path. 2012;8(5). doi: 10.1371/journal.ppat.1002706 22615566 10.1371/journal.ppat.1002706 PMC3355094

[12] Garcia-Mayoral MF, Canales A, Diaz D, Lopez-Prados J, Moussaoui M, de Paz JL, et al Insights into the glycosaminoglycan-mediated cytotoxic mechanism of eosinophil cationic protein revealed by NMR. ACS Chem Biol. 2013;8(1):144–151. doi: 10.1021/cb300386v 23025322 10.1021/cb300386v

[13] Eustermann S, Brockmann C, Mehrotra PV, Yang JC, Loakes D, West SC, et al Solution structures of the two PBZ domains from human APLF and their interaction with poly(ADP-ribose). Nat Struct Mol Biol. 2010;17(2):241–243. doi: 10.1038/nsmb.1747 20098424 10.1038/nsmb.1747 PMC2912505

[14] Schuttelkopf AW, van Aalten DMF. PRODRG: a tool for high-throughput crystallography of protein-ligand complexes. Acta Crystallogr Sect D Biol Crystallogr. 2004;60:1355–1363. doi: 10.1107/S0907444904011679 15272157 10.1107/S0907444904011679

[15] Schallus T, Feher K, Sternberg U, Rybin V, Muhle-Goll C. Analysis of the specific interactions between the lectin domain of malectin and diglucosides. Glycobiology. 2010;20(8):1010–1020. doi: 10.1093/glycob/cwq059 20466650 10.1093/glycob/cwq059

[16] Ghasriani H, Belcourt PJF, Sauve S, Hodgson DJ, Brochu D, Gilbert M, et al A single N-acetylgalactosamine residue at threonine 106 modifies the dynamics and structure of interferon alpha 2a around the glycosylation site. J Biol Chem. 2013;288(1):247–254. doi: 10.1074/jbc.M112.413252 23184955 10.1074/jbc.M112.413252 PMC3537019

[17] De Gonzalo CVG, Zhu LY, Oman TJ, van der Donk WA. NMR structure of the S-linked glycopeptide sublancin 168. ACS Chem Biol. 2014;9(3):796–801. doi: 10.1021/cb4008106 24405370 10.1021/cb4008106 PMC3985867

[18] Borgert A, Heimburg-Molinaro J, Song XZ, Lasanajak Y, Ju TZ, Liu M, et al Deciphering structural elements of mucin glycoprotein recognition. ACS Chem Biol. 2012;7(6):1031–1039. doi: 10.1021/cb300076s 22444368 10.1021/cb300076s PMC3508731

[19] Ulrich EL, Akutsu H, Doreleijers JF, Harano Y, Ioannidis YE, Lin J, et al BioMagResBank. Nucleic Acids Res. 2008;36:D402–D408. doi: 10.1093/nar/gkm957 17984079 10.1093/nar/gkm957 PMC2238925

[20] Lutteke T, Frank M, von der Lieth CW. Carbohydrate Structure Suite (CSS): analysis of carbohydrate 3D structures derived from the PDB. Nucleic Acids Res. 2005;33:D242–D246. doi: 10.1093/nar/gki013 15608187 10.1093/nar/gki013 PMC539967

[21] Hiruma-Shimizu K, Hosoguchi K, Liu Y, Fujitani N, Ohta T, Hinou H, et al Chemical synthesis, folding, and structural insights into O-fucosylated epidermal growth factor-like repeat 12 of mouse Notch-1 receptor. J Am Chem Soc. 2010;132(42):14857–14865. doi: 10.1021/ja105216u 20883017 10.1021/ja105216u

[22] Chaffey PK, Guan XY, Chen C, Ruan Y, Wang XF, Tran AH, et al Structural insight into the stabilizing effect of O-glycosylation. Biochemistry. 2017;56(23):2897–2906. doi: 10.1021/acs.biochem.7b00195 28494147 10.1021/acs.biochem.7b00195

[23] Metzler WJ, Bajorath J, Fenderson W, Shaw SY, Constantine KL, Naemura J, et al Solution structure of human CTLA-4 and delineation of a CD80/CD86 binding site conserved in CD28. Nat Struct Biol. 1997;4(7):527–531. 9228944 10.1038/nsb0797-527

[24] Vakonakis I, Langenhan T, Promel S, Russ A, Campbell ID. Solution structure and sugar-binding mechanism of mouse latrophilin-1 RBL: a 7TM receptor-attached lectin-like domain. Structure. 2008;16(6):944–953. doi: 10.1016/j.str.2008.02.020 18547526 10.1016/j.str.2008.02.020 PMC2430599

